# Concurrent Use of Opioids with Other Central Nervous System-Active Medications Among Older Adults

**DOI:** 10.1089/pop.2019.0128

**Published:** 2020-07-28

**Authors:** Shirley Musich, Shaohung S. Wang, Luke B. Slindee, Joann Ruiz, Charlotte S. Yeh

**Affiliations:** ^1^ Research for Aging Populations, Optum, Ann Arbor, Michigan, USA.; ^2^ Informatics & Data Science, Optum, Minneapolis, Minnesota, USA.; ^3^ Medicare & Retirement, UnitedHealthcare, Minneapolis, Minnesota, USA.; ^4^ AARP Services, Inc., Washington, District of Columbia, USA.

**Keywords:** older adults, opioids, CNS-active medications, injurious falls, emergency room use

## Abstract

The primary objective was to determine the prevalence and characteristics of older adults concurrently using opioids and other central nervous system (CNS)-active medications, and the specialties of providers who ordered the medications. A secondary objective was to document medication-related adverse effects associated with such concurrent drug use. Study populations were identified as older adults aged ≥65 years with 1 year continuous medical and drug plan enrollment during 2017 and opioid use of ≥2 prescriptions for ≥15 days' supply. CNS-active medications included benzodiazepines, non-benzodiazepine hypnotics, muscle relaxants, antipsychotics, and gabapentinoids. Provider specialties were identified from the National Provider Identification database. Characteristics associated with opioids only, opioids plus 1, and opioids plus ≥2 additional CNS-active medications were determined using multinomial logistic regression. Outcome measures during 2017 included injurious falls/fractures and ≥3 emergency room (ER) visits. Among eligible insureds (N = 209,947), 57% used opioids only, 28% used opioids plus 1 additional CNS medication, and 15% used ≥2 additional medications. About 60% of opioids and other concurrent CNS medications were prescribed by the same provider, generally a primary care provider. Benzodiazepines and gabapentinoids were most often used concurrently with opioids. Health status, insomnia, anxiety, depression, and low back pain had the strongest associations with concurrent medication use. Overall, concurrent use with ≥2 CNS medications increased the likelihood of injurious falls/fractures or ≥3 ER visits in this population by about 18% and 21%, respectively. Both patients and providers may benefit from an awareness of adverse outcomes associated with concurrent opioid and other CNS-active medication use.

## Introduction

In 2016, the Centers for Disease Control and Prevention (CDC) issued guidelines for the use of opioids in the management of chronic pain.^1^ One of the 12 key recommendations focused on the concurrent prescribing of opioid pain medications along with other central nervous system (CNS)-active medications, especially benzodiazepines.^1^ The guidelines called for avoidance of concurrent prescribing of opioids and specifically benzodiazepines based on established adverse risks associated with concurrent use, notably respiratory depression, subsequently associated with overdose deaths, altered mental states affecting vehicle safety, and postural stability associated with falls/fractures in the those aged ≥65 years.^1^ The CDC guidelines were supported by the American Geriatrics Society 2019 Updated Beers Criteria for Potentially Inappropriate Medications in Older Adults^2^ and the US Federal Drug & Food Administration Black Box warning of serious risks and death associated with combining opioid pain medications with benzodiazepines.^3^ Benzodiazepines and other CNS-active medications are often used in combination with opioids to augment analgesic effects and/or to manage insomnia, anxiety, and other mental health disorders frequently associated with chronic pain.^4–12^ Yet, despite the contraindications and warnings, concurrent use of opioids with other CNS-active medications, especially benzodiazepines, remains common across age groups and within various patient settings.^8,9,13,14^

To date, concurrent use has no consistent definition in the scientific literature and has been variously defined as an overlap of 1 day,^15–17^ 30 days,^18^ or 90 days^7^ when pharmaceutical claims were available or, more commonly, as during the same time period on surveys such as the National Ambulatory Medical Care Survey.^8,9,13,14,19^ Because the medications are widely utilized across all age groups, study samples are often population based including adults aged ≥18 years with fewer studies focused on older adults aged ≥65 years.^4,8,11,19–21^ Furthermore, there is no consistent list of other CNS-active medications used with opioids. Concurrent use studies have been unilateral, such as those describing opioids with benzodiazepines^7–10,14,17,18,22^ or opioids with gabapentinoids,^12,23,24^ or have included various combinations of opioids with benzodiazepines, non-benzodiazepine hypnotics, antipsychotics, muscle relaxants, and/or gabapentinoids.^4–6,13,15,16,19^ Consequently, the prevalence of concurrent use varies in the scientific literature depending on the age range of study samples, definitions of use, number of medications included, and the time frame of the study. Concurrent use has been reported from about 13% to 49% for opioids combined with a single CNS-active medication,^6–8,10,13,16–18^ such as benzodiazepines, non-benzodiazepine hypnotics, or antipsychotics, and from 25% to 55% for opioids combined with >1 CNS medication.^5,6,15,19^ Such concomitant pharmaceutical solutions are frequently offered as first-line treatment options whether patients present in a provider's office or in the emergency room (ER) for the treatment of chronic pain complicated with anxiety, insomnia, or other mental health issues.^5,6,8,11,14,18,19,22,25^

The providers prescribing concurrent opioids and other CNS-active medications have been characterized primarily as primary care providers (PCPs) who manage the regular care of their patients.^5,6,8,14,18,19^ However, the Veteran's Administration (VA), with a designed specialist referral system and computerized drug monitoring, has reported opioids to be more often prescribed by PCPs, with benzodiazepines and other mental health medications more often prescribed by mental health providers.^5,15,25^ As a result, more than 60% of concurrent use prescriptions were ordered by different providers.^15^ Thus, there is a debate as to whether care fragmentation across multiple providers is a major source of the concurrent use patterns or, in contrast, it is a lack of awareness and appreciation of the risks associated with concurrent use when prescribed by the same providers.^7,15^ When queried in qualitative interviews, physicians stated an awareness of the risks associated with concurrent use but cited a lack of alternatives in managing anxiety, insomnia, and pain.^26,27^ They referenced pharmaceutical management as a cost-efficient approach in preference to referrals to other specialists or hospitalizations, but voiced the need for more non-pharmacological alternatives.^27^

Attention to medication-related adverse effects associated with concurrent use of opioids and other CNS-activity medications has focused, as a priority, on respiratory depression and subsequent overdose deaths.^1,7,9,17,22^ However, in one population-based study, of those who suffered overdose deaths from combined opioids and benzodiazepines, only 2% of those deaths were aged ≥65 years.^22^ The age group with the most deaths was 45–54 years; 55–64 years had the largest percentage increase in deaths.^22^ Consistent with those results, disabled Medicare-eligible patients aged <65 years were more likely to be concurrent users than those ≥65 years (36% vs. 20%).^16^ Thus, although overdose deaths associated with opioids and other CNS medications remain a relevant risk to older adults, altered mental states, postural instability, and possible dizziness resulting in increased falls and fractures may be most concerning.^10,21^ Risk for falls/fractures has been associated with opioids per se (hazard ratio 4.9 fractures upon initiation)^21^ and for combinations of opioids and benzodiazepines (self-reported falls; odds ratio [OR] 3.3).^10^ Subsequent to injury-related ER visits (OR 3.8), increased ER utilization also has been documented among older adults with chronic pain (OR 5.5).^20^ Unmanaged anxiety and panic attacks, sometimes presenting as chest pain, also are common reasons for increased and repeated ER utilization among older adults.^28^

Although recent research studies have considered the prevalence and characteristics associated with concurrent use of opioids with selected CNS-active medications, few have considered an inclusive list of CNS-active medications, used a measured definition of concurrent use or focused on the subpopulation of older adults aged ≥65 year with Medicare Supplement plans (ie, Medigap).^29^ In the United States, government-funded Medicare covers adults aged 65 years and older, as well as those younger than age 65 and disabled. Medicare fee-for-service plans (about 70% of all Medicare plans) pay about 80% of medical expenditures for these individuals but offer minimal prescription drug benefits. Those enrolled in these Medicare plans are personally responsible for obtaining additional insurance plans to cover the remaining 20% of medical expenses (ie, Medicare Supplement or Medigap plans) and more inclusive prescription drug coverage (Medicare Part D plans). Although most (about 90%) of those with original fee-for-service Medicare coverage have some type of supplemental insurance coverage, about 28% (currently about 10.2 million adults) have purchased Medigap coverage.^29^ Because this population may differ in demographic, socioeconomic, or health status characteristics from general older adult and/or specifically overall Medicare populations, it was of interest to examine the prevalence and characteristics associated with the concurrent use of opioids with other CNS-active medications in this study sample and to identify the numbers and specialties of providers ordering the prescriptions for these combinations of drugs. Inherent to a consideration of possible interventions that might enhance compliance with CDC guidelines is an understanding of whether prescriptions come from the same providers (ie, awareness of guidelines) or from different providers (ie, care coordination problem) including the identification of the specialty types (eg, primary care physician, psychiatrist, pain medicine) writing the prescriptions.

Thus, the primary objective was to estimate the prevalence and characteristics associated with concurrent use of opioids with other CNS-active medications in the study sample, along with the identification of the numbers and specialties of providers who were the sources of the various prescriptions. The secondary objective was to consider medication-related adverse outcomes associated with concurrent drug use, especially injurious falls/fractures and utilization of multiple ER visits. This research was reviewed and approved by the New England IRB #120160532.

## Methods

### Study sample

In 2016, approximately 5 million Medicare insureds were covered by an AARP™ Medicare Supplement plan insured by UnitedHealthcare Insurance Company (UnitedHealthcare Insurance Company of New York for New York certificate holders). These plans are offered in all 50 states, Washington, DC, and various US territories. A sample of AARP Medicare Supplement insureds with AARP™ MedicareRx plans insured through UnitedHealthcare (about 55% of insureds) with at least 2 opioid prescriptions and a cumulative day's supply ≥15 days during 2017 was utilized to identify concurrent users of opioids with other CNS-active medications. Additional inclusion criteria for the study sample included: (1) 12-month continuous medical and drug plan enrollment during 2017, (2) provider specialty identification, (3) at least 65 years of age, and (4) exclusion of cancer or hospice patients. The final study sample that met the inclusion criteria included 209,947 insureds.

### Opioid users

Opioids prescribed during 2017 were identified from National Drug Codes (NDCs) as recommended by the 2018 Health Effectiveness Data and Information Set (HEDIS) quality measures associated with opioid use.^30^ Opioid users were defined as those patients with at least 2 prescriptions for opioids and a cumulative days' supply of ≥15 days. Opioids also were descriptively categorized into 6 mutually exclusive categories partially based on US Drug Enforcement Administration opioid drug schedules for acceptability of medical use, and potential for abuse or dependency. Those categories are: (1) long-acting; (2) short-acting, other Schedule II; (3) short-acting, oxycodone; (4) short-acting, hydrocodone; (5) short-acting, Schedule III-IV and nalbuphine; and (6) tramadol.^31^ Days of supply were calculated from prescriptions recorded in the pharmaceutical drug database during calendar year 2017.

### Other CNS-active medications

Other CNS-active medications often prescribed concurrently with opioids in the management of pain, despite contraindications, included benzodiazepines, non-benzodiazepine hypnotics, muscle relaxants, antipsychotics, and gabapentinoids. These drug classes were defined from NDCs.

### Concurrent use definition

Concurrent use of opioids with other CNS-active medications was defined as an overlap by prescription dates and days of supply for at least 30 days for both opioids and at least one of the other CNS medications (benzodiazepines, non-benzodiazepine hypnotics, muscle relaxants, antipsychotics, or gabapentinoids). Summary categories were subsequently defined as: opioid use only (at least 2 opioid prescriptions with a cumulative days' supply ≥15 days); concurrent use of opioids plus 1 additional CNS medication; and concurrent use of opioids plus ≥2 CNS medications. Additional subcategories of the CNS medications were not considered.

### Numbers and specialties of providers

Numbers of providers and specialty types were identified from the National Provider Identifier (NPI) database maintained by the Centers for Medicare & Medicaid Services (CMS).^32^ NPI data are part of a larger repository of provider information within the National Plan & Provider Enumeration System database maintained by CMS. NPI is a unique identification number for covered health care providers. Covered health care providers and all health plans and health care clearinghouses must use the NPI in the administrative and financial transactions adopted under the Health Insurance Portability and Accountability Act. In addition, concurrent prescriptions of opioids with other CNS medications were identified as ordered by the same provider (eg, opioid and benzodiazepine prescriber) or by different providers (eg, opioid prescriber and benzodiazepine prescriber).

### Covariates

Covariates were included to characterize categories of concurrent users of opioids and other CNS-active medications, and to adjust for other risk factors. These covariates included measures of demographics, socioeconomic factors, health status, and other characteristics taken from health plan eligibility and administrative medical claims.

Demographic questions included age and sex. Age groups were defined as: 65–69; 70–74; 75–79; 80–84; and ≥85 years. Geographical location (Northeast, South, Midwest, or West); low (less than 15% nonwhite), medium (15% to 59% nonwhite), and high (≥60% nonwhite) minority areas; and low (<$40,179), medium ($40,179 to <$57,199), and high (≥$57,199) median household income levels were geocoded from zip codes. AARP Medicare Supplement plan types were grouped by cost-sharing levels, including high-level coverage plans with minimal co-payments or deductibles, and all other plans. A measure of health services access was calculated as PCPs per 100,000 capita. Level of medical services utilization from medical claims was calculated as the Hierarchical Condition Category (HCC) score.^33^ The HCC score is used by CMS to risk adjust medical payments across various medical plans according to the health status of the different insured populations. HCC subgroups were defined as follows and utilized to control for health status: HCC scores <0.5; HCC scores 0.5 to <1.2; HCC scores 1.2 to <2.8; and HCC scores ≥2.8.

### Prevalence of medical conditions

Three chronic conditions related to concurrent use were defined from Charlson comorbidity index (CCI) diagnoses codes: chronic obstructive pulmonary disease, dementia, and rheumatoid arthritis. CCI is a measure of the risk of 1-year all-cause mortality attributable to selected comorbidities that also has been shown to be highly predictive of morbidity and health care expenditures.^34^

Three mental health conditions related to concurrent use were defined from Psychiatric Diagnosis Groups diagnoses codes: opioid use disorders (a measure of opioid dependency), major depression, and anxiety disorders.^35^

Musculoskeletal back pain was defined from back pain diagnoses codes after excluding all back pain associated with cancer, trauma, and drug abuse as defined by the HEDIS code specifications.^30^ Back pain diagnoses were documented at any time during the 12-month study period.

Additional possible medication-related adverse effects also were identified from diagnosis codes: pneumonia, respiratory distress, and serotonin syndrome. These potential adverse effects commonly highlighted in the opioid literature were included as associated with concurrent use with these medications, with the expectation that the conditions may be underestimated.

### Injurious falls/hip fractures

Injurious falls requiring medical services or hip fractures, as a combined measure, were defined from suggested HEDIS diagnoses codes.^30^ Falls or hip fractures were documented from these selected diagnoses codes at any time during the 12-month study period. The risk for falls and subsequent fractures has been identified as independently associated with opioids and other CNS medications. The opioid only category was used as the reference, thus any association of increased fall/fractures would be associated with concurrent use with additional CNS medications.

### ER utilization

The researchers did not have cause of ER visits, thus could only estimate the utilization of ER visits over the course of the study period. Based on the distribution and trends across concurrent use categories, multiple ER users were defined as ≥3 visits during 2017 compared with occasional users with 0–2 visits.

### Statistical models

Demographic variables were unilaterally tested across the 3 concurrent use categories using chi-square or *t* tests for categorical or continuous variables, respectively. Characteristics associated with opioids plus 1 and opioids plus ≥2 additional CNS medications compared to opioid only users were determined using multinomial logistic regression models. Depression and anxiety were highly correlated; consequently, separate characteristics and outcomes regression models were developed. Covariates included all of those variables listed in Table 1. Variables with high correlations (eg, >0.5) were dropped from regression models. All analyses were completed using SAS Enterprise Guide Version 7.1 (SAS Institute Inc., Cary, NC, USA).

**Table 1.** Unadjusted Demographic Characteristics for Concurrent Use of Opioids and Other Central Nervous System-Active Medications: Opioids Only, Opioids Plus One, and Opioids Plus ≥Two

	All Mean or %	Opioids only Mean or %	Opioids plus 1 Mean or %	Opioids plus ≥2 Mean or %
Number	209,947	119,744	58,463	31,740
Sex				
Male	31.0	33.5	28.9	25.3
Female	69.0	66.5	71.1	74.7
Age (years)	75.6	75.9	75.8	74.2
65–69	25.7	24.6	24.9	31.6
70–74	26.8	26.3	26.3	29.3
75–79	19.6	19.8	19.9	18.2
80–85	12.4	12.7	13.1	10.3
≥85	15.5	16.6	15.9	10.6
Minority (from zip codes)				
Low	48.1	48.6	48.0	46.8
Medium	46.9	46.3	47.3	48.7
High	3.4	3.6	3.3	3.1
Median Income (from zip codes)				
Low	18.0	17.2	18.9	19.1
Medium	38.1	37.6	38.9	38.7
High	43.6	44.8	41.9	42.0
Region				
Midwest	17.5	18.1	17.4	15.7
Northeast	16.3	17.3	15.6	13.9
South	43.6	41.9	44.5	48.1
West	22.3	22.4	22.3	22.1
Access to health care				
PCP per 100,000 capita	131.0	131.7	130.6	129.3
Plan type				
High	78.0	77.4	78.1	80.1
Medium	2.9	2.9	3.1	2.7
Other	19.1	19.7	18.9	17.2
HCC Score				
≤0.50	19.4	21.9	16.6	15.2
0.50 to <1.20	40.1	41.9	38.6	36.0
1.20 to <2.80	31.9	29.4	34.4	36.9
≥2.8	8.6	6.8	10.4	12.0
CCI Conditions				
Dementia	10.2	8.8	11.0	14.2
Chronic obstructive pulmonary disease	30.2	27.7	32.1	36.3
Rheumatoid arthritis	8.8	8.0	9.6	10.6
PDG Conditions				
Opioid use disorders	9.2	6.9	10.5	15.2
Major depression	21.3	16.3	23.2	36.6
Anxiety disorders	22.0	14.9	26.0	41.7
Insomnia dx	11.0	6.6	13.4	23.4
Pneumonia dx	10.3	8.9	11.2	13.7
Respiratory distress dx	28.9	27.1	30.2	33.3
Serotonin syndrome dx	0.5	0.4	0.6	1.0
Low back pain dx	46.4	41.5	51.0	56.3
Injury fall/hip fracture dx	11.8	11.6	11.3	13.4
Hip fracture dx	2.4	2.5	2.3	2.3
Injurious fall dx	10.8	10.6	10.4	12.4
Any inpatient hospitalization	32.3	33.2	29.8	33.4
Number of ER visits				
0	53.5	54.0	54.8	49.1
1–2	33.8	34.2	32.6	34.1
≥3	12.8	11.8	12.6	16.7
Opioid category				
Long acting	9.6	5.4	12.6	20.1
Short acting, other Schedule II	5.1	3.9	5.5	8.8
Short acting, oxycodone	31.7	30.8	30.1	38.0
Short acting, hydrocodone	49.2	48.8	48.7	51.6
Short acting, Schedule III–IV	9.7	10.9	8.3	8.0
Tramadol	46.5	49.9	44.6	37.1
Concurrent use of opioids with other CNS medications				
Opioids and gabapentinoids	20.8	0.0	38.8	66.0
Opioids and benzodiazepines	18.4	0.0	31.9	63.1
Opioids and non-benzo hypnotics	12.3	0.0	17.5	49.3
Opioids and muscle relaxants	7.8	0.0	8.7	35.7
Opioids and antipsychotics	3.1	0.0	3.1	14.5

Notes: All variables are statistically significant at *P* < 0.0001. Missing categories deleted for brevity.

CCI, Charlson comorbidity index; CNS, central nervous system; dx, diagnoses codes; ER, emergency room; HCC, Hierarchical Condition Category; PCP, primary care provider; PDG, Psychiatric Diagnosis Groups.

### Sensitivity analyses

As a sensitivity analysis, the researchers also considered a 2-year continuous enrollment study sample 2016–2017 (N = 195,388) using 2016 as a baseline year to identify new and continuing users of opioids and/or CNS medications during 2017. New users could not have any prescriptions for the drugs under study during calendar year 2016. If there were prescriptions prior to 2017, patients were considered as continuing users. Identifying numbers and specialties of providers for new and continuing users were used to confirm any differences in the patterns and provider types for concurrent use prescriptions.

## Results

Overall, 31% of AARP Medicare Supplement insureds filled at least 1 opioid prescription in 2017 and, among these, 50% used at least 2 prescriptions of opioids for ≥15 days (N = 350,936). From this initial study sample, 9% (N = 31,433) were excluded because of continuous enrollment criteria; 27% (N = 95,436) were excluded because of cancer diagnoses or hospice care; 3% (N = 8968) were excluded for age <65 years or missing sex; and 1% (N = 5152) were excluded because of missing provider specialty data. After these exclusions, the final study population included 209,947 insureds (30% of opioid users) in the following concurrent use categories: 57% opioids only; 28% opioids plus 1 additional CNS medication; 15% opioids plus ≥2 additional CNS medications (Table 1). In sensitivity analyses, 29% (N = 56,415) of the 2-year sample were considered new users of opioids (at least 2 prescriptions for ≥15 days) and/or other CNS medications. Of these, 14% used opioids plus 1 and 5% used opioids plus ≥2 additional CNS medications in their first year of drug exposure. Because the results for new and continuing users were similar to the 1-year sample, the researchers opted to show only the results using the 2017 one-year study sample with relevant comparisons noted in the text.

As a group, those with at least 2 opioid prescriptions lasting ≥15 days were mostly female, 70–74 years of age, white, high income, lived in the South region, and were in high-coverage medical plans (Table 1). The most common opioid prescriptions were for short-acting hydrocodone, tramadol, or short-acting oxycodone. Overall, CNS medications used most often in combination with opioids were gabapentinoids, benzodiazepines, and non-benzodiazepine hypnotics. Likewise, for opioids plus 1 or opioids plus ≥2 additional CNS medications, gabapentinoids and benzodiazepines were the most commonly used prescriptions in combination with opioids.

### Numbers and specialties of providers

Generally, about 60% (range 43% to 68%) of concurrent opioids and CNS medications were prescribed by the same provider, most often a PCP (ie, family medicine, internal medicine, nurse practitioner) (Table 2). For the remaining 40% with opioids and CNS medication prescriptions ordered by different providers, the pattern was similar with the exception of pain medicine for opioids and psychiatry for CNS medications (Table 3). Nevertheless, the majority of prescriptions for both opioids and CNS medications, despite being sourced from different providers, were ordered by PCPs.

**Table 2.** Concurrent Opioids and Other Central Nervous System-Active Medications Provider Type Prescribers: Same Provider

Provider type prescriber	Same provider %
**Opioids & Benzodiazepines (N = 23,203)**	**60**
Family Medicine	38
Internal Medicine	38
Nurse Practitioner	6
All others	19
**Opioids & Gabapentinoids (N = 27,013)**	**62**
Family Medicine	30
Internal Medicine	28
Pain Medicine	11
Nurse Practitioner	8
Physician Assistant	5
All others	19
**Opioids & Non-Benzodiazepine Hypnotics (N = 14,303)**	**55**
Family Medicine	38
Internal Medicine	37
Nurse Practitioner	6
All others	19
**Opioids & Muscle Relaxants (N = 11,119)**	**68**
Family Medicine	26
Internal Medicine	20
Pain Medicine	16
Nurse Practitioner	9
Physician Assistant	8
All others	20
**Opioids & Antipsychotics (N = 2762)**	**43**
Family Medicine	38
Internal Medicine	35
Nurse Practitioner	8
Geriatric Medicine	5
All others	14

Notes: All others includes summed total of all other provider types individually less than 5% of prescriptions.

**Table 3.** Concurrent Opioids and Other Central Nervous System-Active Medications Provider Type Prescribers: Different Providers

Provider type prescribers	Different provider %	Provider type prescribers	Different provider %
**Opioids (N = 15,447)**	**40**	**Benzodiazepines**	**40**
Pain Medicine	22	Internal Medicine	29
Family Medicine	12	Family Medicine	27
Internal Medicine	12	Psychiatry	18
Nurse Practitioner	10	Nurse Practitioner	7
Physician Assistant	9	All others	19
Orthopedic Surgery	7		
Physical Medicine & Rehab	6		
Rheumatology	6		
All others	17		
**Opioids (N = 16,631)**	**38**	**Gabapentinoids**	**38**
Pain Medicine	18	Internal Medicine	22
Family Medicine	16	Family Medicine	21
Internal Medicine	16	Nurse Practitioner	12
Nurse Practitioner	11	Neurology	11
Physician Assistant	9	Physician Assistant	7
Orthopedic Surgery	6	Pain Medicine	5
All others	25	All others	22
**Opioids (N = 11,566)**	**45**	**Non-Benzodiazepine Hypnotics**	**45**
Pain Medicine	22	Internal Medicine	31
Family Medicine	11	Family Medicine	29
Internal Medicine	11	Psychiatry	14
Nurse Practitioner	10	Nurse Practitioner	8
Physician Assistant	9	All others	18
Orthopedic Surgery	8		
Physical Medicine & Rehab	6		
Rheumatology	6		
All others	16		
**Opioids (N = 5304)**	**32**	**Muscle Relaxants**	**32**
Pain Medicine	22	Family Medicine	21
Family Medicine	17	Internal Medicine	18
Internal Medicine	14	Nurse Practitioner	17
Nurse Practitioner	11	Physician Assistant	10
Physician Assistant	8	Neurology	9
Orthopedic Surgery	5	Pain Medicine	6
All others	24	Rheumatology	5
		All others	14
**Opioids (N = 3668)**	**57**	**Antipsychotics**	**57**
Internal Medicine	18	Psychiatry	45
Pain Medicine	17	Internal Medicine	16
Family Medicine	16	Family Medicine	14
Nurse Practitioner	14	Nurse Practitioner	7
Physician Assistant	7	Neurology	6
All others	28	All others	12

Notes: All others includes summed total of all other provider types individually less than 5% of prescriptions.

In sensitivity analyses, numbers and specialty types of providers were similar for both new and continuing users, reflecting the conclusions of the 1-year study sample. New users of concurrent drugs had a majority of prescriptions ordered by the same providers (range 41% to 65%), most often a PCP. Thus, concurrent prescriptions appear to be prescribed by PCPs managing the various physical and mental health symptoms of their patients, without referrals to other specialties.

### Characteristics associated with opioids plus 1 and opioids plus ≥2 CNS medications

The characteristics with the strongest associations with concurrent use of opioids with CNS medications were poor health status (high HCC scores); mental health conditions of insomnia, anxiety and depression; and youngest age group 65–69 years (Table 4). Other significant characteristics of note associated with both levels of concurrent drug use included female, low minority (white), low income, diagnosed low back pain, and more likely to experience an injurious fall/fracture (opioids plus ≥2 only).

**Table 4.** Characteristics Associated with Opioids Plus One and Opioids Plus ≥Two Additional Central Nervous System-Active Medications Compared to Opioids Only

Variable	Depression models				Anxiety models			
	Opioids plus 1		Opioids plus ≥2		Opioids plus 1		Opioids plus ≥2	
	Odds ratio	P value	Odds ratio	P value	Odds ratio	P value	Odds ratio	P value
Female	1.31	<0.0001	1.63	<0.0001	1.27	<0.0001	1.56	<0.0001
Age 65–69	1.26	<0.0001	2.67	<0.0001	1.26	<0.0001	2.69	<0.0001
Age 70–74	1.17	<0.0001	2.07	<0.0001	1.16	<0.0001	2.08	<0.0001
Age 75–79	1.11	<0.0001	1.59	<0.0001	1.11	<0.0001	1.59	<0.0001
Age 80–84	1.07	0.0003	1.26	<0.0001	1.07	0.0009	1.25	<0.0001
Minority low	1.14	<0.0001	1.18	<0.0001	1.13	<0.0001	1.15	<0.0001
Minority medium	1.16	<0.0001	1.22	<0.0001	1.14	<0.0001	1.20	<0.0001
Income low	1.16	<0.0001	1.13	<0.0001	1.15	<0.0001	1.11	<0.0001
Income middle	1.09	<0.0001	1.05	0.004	1.08	<0.0001	1.04	0.01
Midwest	0.93	<0.0001	0.80	<0.0001	0.93	<0.0001	0.80	<0.0001
Northeast	0.90	<0.0001	0.76	<0.0001	0.89	<0.0001	0.75	<0.0001
West	0.96	0.005	0.86	<0.0001	0.98	0.13	0.89	<0.0001
Plan type: medium	1.07	0.04	0.95	0.22	1.07	0.03	0.96	0.35
Plan type: others	0.99	0.65	0.95	0.007	1.00	0.75	0.96	0.01
PCP per 100,000 capita	1.00	0.003	1.00	<0.0001	1.00	0.005	1.00	<0.0001
HCC Score 0.50 to <1.20	1.28	<0.0001	1.51	<0.0001	1.28	<0.0001	1.55	<0.0001
HCC Score 1.20 to <2.80	1.66	<0.0001	2.34	<0.0001	1.67	<0.0001	2.44	<0.0001
HCC Score ≥2.8	2.21	<0.0001	3.32	<0.0001	2.23	<0.0001	3.53	<0.0001
Rheumatoid arthritis dx	1.08	<0.0001	1.09	0.0003	1.09	<0.0001	1.10	<0.0001
Opioid use disorders	1.41	<0.0001	1.79	<0.0001	1.37	<0.0001	1.71	<0.0001
Depression dx	1.37	<0.0001	2.27	<0.0001	-	-	-	-
Anxiety dx	-	-	-	-	1.80	<0.0001	3.21	<0.0001
Insomnia dx	2.06	<0.0001	3.70	<0.0001	1.97	<0.0001	3.46	<0.0001
Low back pain dx	1.49	<0.0001	1.86	<0.0001	1.48	<0.0001	1.83	<0.0001
Injurious falls/fractures dx	0.93	<0.0001	1.12	<0.0001	0.92	<0.0001	1.11	<0.0001

Notes: Reference categories include: male; age ≥85; minority high; income high; South; plan type: high; HCC Score <0.50; no rheumatoid arthritis; no opioid use disorders; no depression; no anxiety; no insomnia; no low back pain, and no injurious falls/fractures.

dx, diagnosis code; HCC, Hierarchical Condition Category; PCP, primary care provider.

### Adjusted injurious falls/fractures and multiple ER utilization

Opioids plus 1 additional CNS medication had minimal/no association with increased injurious falls/fractures or multiple (≥3 visits) ER utilization. However, concurrent use of opioids plus ≥2 additional CNS medications, adjusted for the variables in Table 1, maintained a significant association with both increased injurious falls/fractures and increased multiple ER utilization: by 18% and 21%, respectively (depression models), and by 18% and 14%, respectively (anxiety models) (Tables 5 and 6).

**Table 5.** Adjusted Odds Ratios for Injurious Falls/Fractures Associated with Opioids and Other Central Nervous System-Active Medications: Plus One and Plus ≥Two

Variable	Depression model Injurious falls/fractures		Anxiety model Injurious falls/fractures	
	Odds ratio	P value	Odds ratio	P value
**Opioids plus 1 CNS medication**	0.96	0.02	0.95	0.003
**Opioids plus ≥2 CNS medications**	1.18	<0.0001	1.18	<0.0001
Female	1.30	<0.0001	1.31	<0.0001
Age 70–74	1.35	<0.0001	1.34	<0.0001
Age 75–79	1.80	<0.0001	1.79	<0.0001
Age 80–84	2.62	<0.0001	2.60	<0.0001
Age ≥85	3.45	<0.0001	3.43	<0.0001
Minority low	1.09	0.02	1.09	0.02
Minority medium	1.07	0.06	1.07	0.05
Income low	1.02	0.35	1.00	0.97
Income middle	0.99	0.40	0.98	0.21
Midwest	1.12	<0.0001	1.14	<0.0001
Northeast	1.17	<0.0001	1.17	<0.0001
West	0.99	0.69	1.01	0.61
Plan type: medium	0.83	<0.0001	0.83	<0.0001
Plan type: others	0.93	0.0002	0.93	<0.0001
PCP per 100,000 capita	1.00	<0.0001	1.00	<0.0001
Rheumatoid arthritis dx	1.16	<0.0001	1.19	<0.0001
Opioid use disorders	1.54	<0.0001	1.56	<0.0001
Depression dx	2.09	<0.0001	-	-
Anxiety dx	-	-	1.74	<0.0001
Insomnia dx	1.31	<0.0001	1.33	<0.0001
Low back pain dx	0.36	<0.0001	0.35	<0.0001

Notes: Reference categories included: male; age 65–69; minority high; income high; South; plan type: high; no rheumatoid arthritis; no opioid use disorders; no depression; no anxiety; no insomnia; and no low back pain.

CNS, central nervous system; dx, diagnosis code; PCP, primary care provider.

**Table 6.** Adjusted Odds Ratios for Three or More Emergency Room Visits Associated with Opioids and Other Central Nervous System-Active Medications: Plus One and Plus ≥Two

Variable	Depression models ≥3 ER visits		Anxiety models ≥3 ER visits	
	Odds ratio	P value	Odds ratio	P value
**Opioid plus 1 CNS medication**	0.97	0.08	0.94	<0.0001
**Opioid plus ≥2 CNS medications**	1.21	<0.0001	1.14	<0.0001
Female	0.97	0.03	0.95	0.002
Age 70–74	1.18	<0.0001	1.18	<0.0001
Age 75–79	1.48	<0.0001	1.48	<0.0001
Age 80–84	1.88	<0.0001	1.88	<0.0001
Age ≥85	2.14	<0.0001	2.15	<0.0001
Minority low	0.90	0.001	0.89	0.0003
Minority medium	0.91	0.004	0.91	0.003
Income low	1.05	0.02	1.03	0.20
Income middle	1.04	0.009	1.03	0.03
Midwest	1.04	0.03	1.06	0.004
Northeast	0.99	0.64	0.99	0.56
West	1.04	0.04	1.07	0.0002
Plan type: medium	0.90	0.007	0.90	0.009
Plan type: others	0.91	<0.0001	0.91	<0.0001
PCP per 100,000 capita	1.00	0.003	1.00	0.01
Rheumatoid arthritis dx	1.35	<0.0001	1.38	<0.0001
Opioid use disorders	1.98	<0.0001	1.96	<0.0001
Depression dx	2.24	<0.0001	-	-
Anxiety dx	-	-	2.30	<0.0001
Insomnia dx	1.40	<0.0001	1.37	<0.0001
Low back pain dx	0.84	<0.0001	0.83	<0.0001

Notes: Reference categories included: male; age 65–69; minority high; income high; South; plan type: high; no rheumatoid arthritis; no opioid use disorders; no depression; no anxiety; no insomnia; and no low back pain.

CNS, central nervous system; dx, diagnosis code; ER, emergency room; PCP, primary care provider.

In sensitivity analyses, injurious falls/fractures and multiple ER utilization for continuing users were increased significantly for opioids plus ≥2 additional CNS medications by 33% and 33%, respectively, and by 45% and 39%, respectively, for new users. Opioids plus 1 CNS medication, although statistically significant in the 2-year models, was minimally associated with either of the adverse outcomes (1% to 7% increased).

## Discussion

In this study sample of AARP Medicare Supplement insureds with extended opioid use, 57% used opioids only, 28% used opioids plus 1 additional CNS medication, and 15% used ≥2 additional medications. Although definitions of “concurrent use” for opioids and other CNS-active medications varied and fewer studies focused exclusively on study populations aged ≥65 years, the prevalence of those in this study with documented concurrent use of ≥30 days was in general agreement with previous publications (ie, about 20% concurrent use for a single medication; about 40% for ≥2).^5–8,10,13,15–19^ As in other studies, those who used opioids and other CNS medications concurrently often were taking other medications to manage mental health problems of anxiety, insomnia, or depression associated with their chronic pain management.^4–11^ Not surprisingly, the most commonly used concurrent medications with opioids were gabapentinoids and benzodiazepines.^6,12,15,19,25^ Use of gabapentinoids has increased dramatically in recent years to augment the analgesic effect of opioids, perhaps associated with pressure to use lower dosages of opioids.^12,24^

About 60% of opioids and other CNS-active medications were prescribed by the same providers, most often a PCP. Thus, although care fragmentation with multiple prescriptions ordered across multiple providers may contribute to the problem,^7,15^ these data would suggest that PCPs managing the regular care of their patients are the source of much concurrent prescribing despite guidelines and warnings to the contrary. It would appear that whether patients present in the PCP office or the ER, providers are asked to address pain and mental health-related symptoms for which there are few alternatives.^5,6,9,14,18,19,22,25–27^ Contrary to the VA system, with drug-monitoring systems and a referral system to other specialists,^5,10,15,26^ the general PCP has no such resources. Thus, pharmaceutical solutions remain first-line treatment options in the general care of broader populations of adults to manage pain along with anxiety, insomnia, depression and the multiple chronic conditions common among older adults.^4,5,8,14,18,19,25^

Characteristics associated with concurrent use of opioids and other CNS medications included anxiety, insomnia, depression, low back pain, and poorer health, but younger age groups 65–69 years. Other characteristics of note included female, white, lower income, and more likely to suffer an injurious fall/fracture. These characteristics are generally consistent across research studies.^4–11,13,14,17^ That concurrent use of medication increased with younger age groups is noteworthy as these individuals age into Medicare-eligible coverage plans. Problems with anxiety, depression, and insomnia have been consistently associated with pain management but to date have few alternatives to pharmacological treatments.^4–11,27^

Medication-related adverse effects measured in this study included respiratory distress, pneumonia, serotonin syndrome, and injurious fall/fractures. Of these conditions, only injurious falls/fractures demonstrated a significant association with concurrent use of opioids plus ≥2 CNS medications. The magnitude of increased injurious falls/fractures in the present study, about 20%, was less than the parallel Yorborough et al study^10^ that reported more than 3 times higher falls among concurrent users. Their study on a younger study sample (59 years) used self-reported falls as the outcome, which may explain the difference in part. In the present study sensitivity analysis, new users of these drugs had increased falls/fractures by about 50%. This increased likelihood is consistent with suggestions that the risk of falls/fractures is comparatively highest upon initiation and dissipates somewhat over time.^21^ The researchers did not know the reasons associated with ER utilization, and hence could not track drug-drug interactions or specific anxiety-related visits. But, if one assumes that CNS-active medication treatments are consistent with the documented diagnoses of anxiety, insomnia, and depression, the 20% increased multiple ER visits would be consistent with other studies, and with the suggestion that many avoidable ER visits are associated with anxiety and pain-related symptomology.^10,14,17,20,25,27^

At the provider level, suggestions have been made for improved education and policy changes to guide providers in avoiding concurrent use of these medications.^7,14,23,27^ Better integration of pharmacists with access to prescription monitoring systems could help alert providers of drug overlaps and interactions, especially from different providers. More available referral patterns could provide PCPs access to pain medicine and mental health specialists as options for patient care.^5,6,14,18,19,25^ Perhaps, most importantly, there are few non-pharmacological alternatives available for the treatment of anxiety, insomnia, and chronic pain issues. Non-pharmacological approaches have shown success among older adults and include: cognitive-behavioral therapies (CBT) for anxiety, insomnia, and/or pain; mindfulness; acupuncture; and physical therapy options.^14,18,19,36–38^ In the PCP environment, however, multi-session interventions have lacked practicality because of time and resource limitations. In addition, many published interventions remain within research settings, involve small study samples, and consequently have not been widely implemented.

Direct-to-patient communications also have been suggested to inform patients of the risks associated with these medication combinations relative to the severity of their symptoms.^19,26^ Once patients have concurrent prescriptions, safe tapering of opioids and/or CNS mediations requires patient–provider agreement, a stepwise, time-consuming protocol, and, although demonstrating some success, has been characterized by a high recidivism rate.^39^ In the VA environment, depression management among noncancer pain patients has been associated with opioid cessation.^40^ Finally, health plans, including Medicare and/or Medicare Advantage, may need to consider more inclusive coverage to include more mental health benefits, more non-pharmacological options such as CBT, and better access to pain and mental health specialists.^9^

### Limitations

This study has some limitations. The study population of AARP Medicare Supplement insureds may not generalize to all older adults or other Medicare, Medicare Advantage or Medicare Supplement beneficiaries. Pharmacy databases confirmed prescription purchases but there was no indication of whether patients actually consumed the drugs as directed. Mental health issues were identified from diagnosis codes and/or medication use, thus depression, insomnia and anxiety were likely underreported. Medication-related adverse effects were identified from diagnosis codes and may be underestimated. Strengths of the study included a large study population that applied a measured definition of 30 days of overlap for concurrent use of a more inclusive list of CNS medications. Provider specialties were documented from the external NPI database. Characteristics of concurrent medication users and selected medication-related adverse effects of falls/fractures and multiple ER use provided comprehensive information to define the opioid and CNS medication concurrent use problem in consideration of possible solutions.

## Conclusions

Overall, in this study sample of Medicare Supplement insureds with extended opioid use, about 40% used ≥1 concurrent CNS-active medications. Anxiety, depression, and insomnia were prominent mental health conditions associated with such use. Contrary to expectations, prescriptions were most often from the same provider, generally a PCP. Increased falls/fractures and multiple ER use were associated with concurrent use of ≥2 CNS medications. Better awareness of the adverse effects associated with concurrent opioid and other CNS medication use may benefit both patients and providers. More evidence-based non-pharmacological options to address anxiety, insomnia, and pain symptoms are warranted.
